# Mass Spectrometric Analysis of Clomiphene Citrate-induced Changes in the Secretome Profile of PAX8-positive Human Fallopian Tube Secretory Epithelial Cells and Identification of Biomarkers Relevant to Reproduction and Pregnancy

**DOI:** 10.1007/s43032-025-01884-w

**Published:** 2025-06-11

**Authors:** Opalina Roy, Shruthi Ammankallu, Vanya Kadla Narayana, Chinmaya Narayana, Shripad Hebbar, Prashanth K. Adiga, Manjunath B. Joshi, Jeevan Gowda, Anujith Kumar, Satish Kumar Adiga, Megan Ritting, Nagarajan Kannan, Thottethodi Subrahmanya Keshava Prasad, Guruprasad Kalthur

**Affiliations:** 1 Department of Reproductive Science, Kasturba Medical College, Manipal Academy of Higher Education, Manipal, 576104 Karnataka India; 2 Center for Systems Biology and Molecular Medicine [an ICMR-Collaborating Centre of Excellence (ICMR-CCoE 2024)], Yenepoya Research Centre, Yenepoya (Deemed to Be University), Mangalore, 575018 India; 3 Department of Obstetrics and Gynecology, Kasturba Medical College, Manipal Academy of Higher Education, Manipal, 576104 Karnataka India; 4 Department of Reproductive Medicine and Surgery, Kasturba Medical College, Manipal Academy of Higher Education, Manipal, 576104 Karnataka India; 5 Department of Ageing Research, Manipal School of Life Sciences, Manipal Academy of Higher Education, Manipal, 576104 Karnataka India; 6 Manipal Institute of Regenerative Medicine, Manipal Academy of Higher Education, Bangalore, 560065 Karnataka India; 7 Stem Cell and Cancer Biology Laboratory, Mayo Clinic, Rochester, MN 55905 USA; 8 Department of Laboratory Medicine and Pathology, Mayo Clinic, Rochester, MN 55905 USA; 9 Center for Regenerative Biotherapeutics, Mayo Clinic, Rochester, MN 55905 USA; 10 Mayo Clinic Comprehensive Cancer Center, Mayo Clinic, Rochester, MN 55905 USA

**Keywords:** Infertility, Ovulation induction, Ectopic pregnancy, Selective estrogen receptor modulator (SERM), Spermatozoa

## Abstract

Clomiphene citrate (CC) is commonly used for infertility treatments, particularly in intrauterine insemination (IUI) and in vitro fertilization (IVF) programs. Despite its effectiveness in inducing ovulation, it is reported to have a low pregnancy rate compared to letrozole. Further, risks of ectopic pregnancy, miscarriage, and fetal malformations have been reported in CC-induced fertility treatment cycles. The present study aimed at understanding whether these effects are associated with CC-induced changes in the secretome profile of the human PAX8-positive fallopian tube secretory epithelial cells (hPFTSECs). Fallopian tube tissues from healthy women who underwent postpartum tubectomy were enzymatically dissociated and cultured with CC, and their secretomes were analyzed by Liquid Chromatography Tandem Mass Spectrometry (LC–MS/MS) to identify CC-induced alterations. Proteomic analysis identified a total of 1549 proteins in the secretome pool of hPFTSECs cultured in vitro and 258 proteins were found to be differentially abundant in response to CC. KEGG and REACTOME analysis revealed that the differentially abundant proteins (DAPs) were mainly associated with pathways such as estrogen signaling, cell cycle and DNA damage. Further, candidate proteins associated with ovulation, embryo development, embryo implantation, pregnancy complications, ectopic pregnancy, sperm functions, apoptosis, senescence, and ovarian cancers were identified. CC exposure induced changes in secretome profile of hPFTSECs may affect the female reproductive health by potentially altering the tubal microenvironment. The study was registered in the Clinical Trials Registry of India (CTRI) under the registration number CTRI/2020/11/028791 on 02/11/2020.

**Supplementary Information:**

The online version contains supplementary material available at 10.1007/s43032-025-01884-w.

## Introduction

Clomiphene citrate (CC), a non-steroidal selective estrogen receptor modulator (SERM), is considered the first-line drug for infertility treatments of women undergoing intrauterine insemination (IUI) and in vitro fertilization (IVF) programs involving mild ovarian stimulation [1]. Comparative analysis with letrozole, an aromatase inhibitor, revealed that even though CC is a good ovulation induction agent, the pregnancy rate is relatively low [2, 3]. Further, higher incidence of ectopic pregnancy [4–6], miscarriage rate [7–9], and moderate risk of fetal malformations [10] over other drugs warrant in-depth research on understanding the complex action of CC on the female reproductive system.

The fallopian tube is an important organ that plays a critical role in oocyte transport, sperm storage, embryo transport and creates a conducive environment for fertilization and pre-implantation embryo development [11]. Hence, any abnormality in the structure and/or function of the fallopian tube has the potential to lead to fertilization failure and infertility. Although CC has limited dose-dependent side effects, long-term treatment is known to be associated with increased chances of ectopic pregnancy [4–6]. Previous studies using rat models have documented tubal dysplasia and ectopic pregnancy due to CC administration [12, 13]. However, there are no available reports to elucidate the effect of CC on human fallopian function, either in vitro or in vivo. Additionally, the detailed mechanism, changes in the function of tubal epithelial cells, and the alteration in the tubal microenvironment remain poorly understood.

A secretome refers to the complete set of proteins secreted by cells or tissues and analyzing its protein composition can reveal minute changes within the microenvironment. Proteomic and metabolomic analysis of secretomes and body fluids has been a popular approach to identify altered secreted proteins and novel biomarkers in the context of cancer [14], polycystic ovary syndrome (PCOS) [15], and infections [16], among others. Proteomic profiling of the secreted proteins in the oviductal fluid detected diverse pathways that define the oviductal microenvironment [17]. Previous proteomic studies in the human fallopian tube have mainly focused on pathological aspects [18–20]. However, no reports documented the consequences of the commonly used ovulation induction drug CC on the secretome profile of hPFTSECs. In this study, we aimed to understand the effect of CC on the function of hPFTSECs to gain deeper insight into the possible alterations of the tubal microenvironment induced by CC.

## Materials and Methods

### Fallopian Tube Tissue Collection

**Fig. 1:**
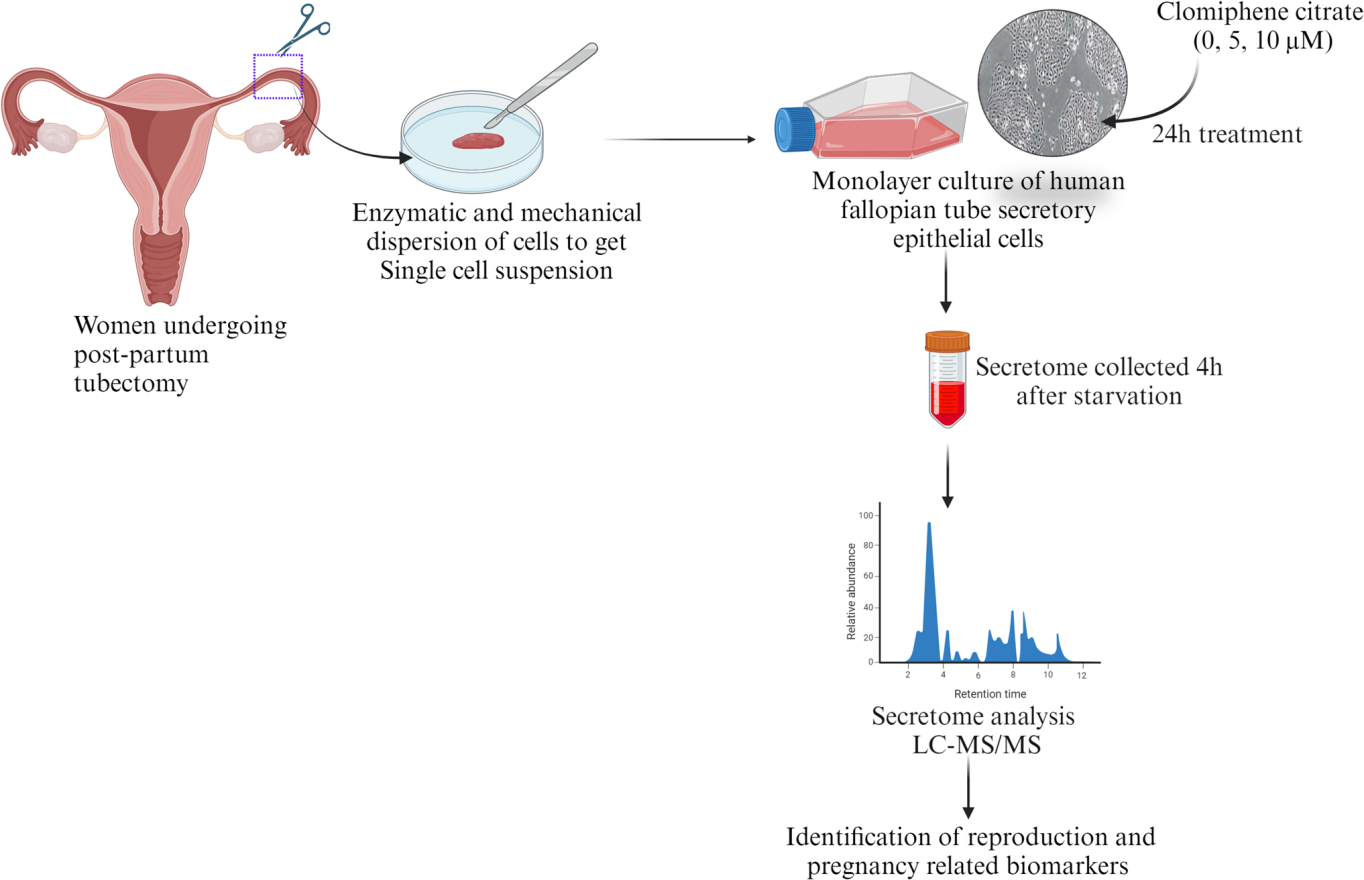
Schematic representation of the study outline to assess the proteomic analysis of the secretome collected from hPFTSECs exposed to clomiphene citrate (CC). Created with BioRender.com

The study was approved by the Institutional Ethics Committee (IEC), Kasturba Medical College, and Kasturba Hospital, Manipal, India (IEC: 332/2020). Tissue specimens from the isthmus region of the fallopian tube were obtained from 5 healthy Indian women aged 20 to 40 years, who underwent postpartum tubectomy for family planning, at the Department of Obstetrics and Gynecology, Kasturba Hospital, Manipal. Upon surgical excision, the fallopian tube samples were immersed in sterile phosphate-buffered saline (PBS) and transported on ice. Written consent was obtained from all the patients participating in the study (Fig. 1).

### Culturing of Human PAX8-positive Fallopian Tube Secretory Epithelial Cells (hPFTSECs)

**Fig. 2:**
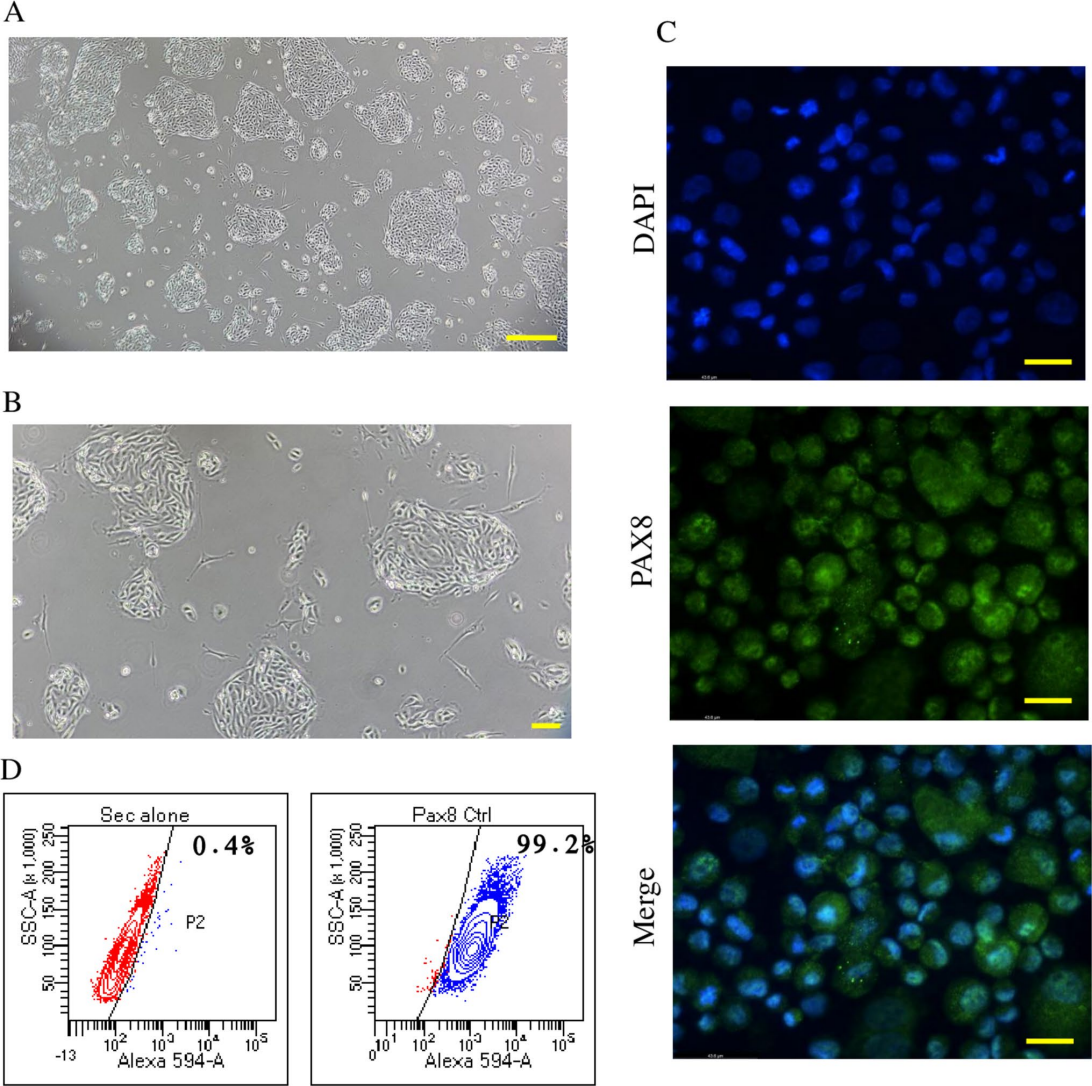
Representative image of primary human fallopian tube secretory epithelial cells in culture at passage-0 showing the typical nest-like cell “islands”. **A** At 40 × magnification. The scale bar represents 400 µm. **B** At 100 × magnification. The scale bar represents 100 µm. Confirmation of the secretory nature of the isolated human fallopian tube epithelial cells by assessing PAX8 expression. **C** Representative images of human fallopian tube secretory epithelial cells expressing PAX8 by immunofluorescence (400 × magnification). The scale bar represents 25 µm. **D** Flow cytometry scatter plot showing PAX8 expression in human fallopian tube secretory epithelial cells by FACS analysis

The fallopian tube tissue was rinsed with PBS to remove any residual blood. Using sterile forceps, the tissue was transferred to a 90-mm Petri dish (Tarsons Cat# 460,090). With a scalpel, visible connective tissue was trimmed and discarded. The tissue was finely minced and transferred to a 50 mL centrifuge tube (Tarsons Cat# 546,041) containing 5 mL of dissociation medium composed of trypsin (1.0 mg/mL) (HiMedia Cat# RM713-25G) and collagenase (1.0 mg/mL, Sigma-Aldrich Cat# C4-22-1G). The tissue mixture was incubated at 37 °C for 2 h with constant agitation to enzymatically release epithelial cells from the bulk tissue. After digestion, the enzyme activity was neutralized by adding Dulbecco’s Modified Eagle Medium/Nutrient Mixture F-12 (DMEM/F12; Thermo Scientific Cat# 11,330,032) supplemented with 10% fetal calf serum (FCS; Thermo Scientific Cat# 10,270,106). The resulting cell suspension was filtered through a 40 µm cell strainer (Sigma-Aldrich Cat# CLS431750-50EA) to remove tissue debris. The filtered suspension was centrifuged at 1000 rpm for 5 min. The supernatant was carefully discarded, and the resulting cell pellet was resuspended in 1 mL of culture medium. To break up any remaining cell clumps, the suspension was gently pipetted up and down at least 10 times using a 1 mL serological pipette. The number of secretory (non-ciliated) epithelial cells was counted using a hemocytometer. Ciliated cells (identified by beating cilia) and red blood cells were excluded. For cell clumps (typically 5–10 cells), the number of secretory cells within each cluster was estimated. The cells were seeded into a T-25 culture flask (Tarsons Cat# 950,010) containing 4 mL of culture medium: DMEM/F12 supplemented with 10% FCS, 10 ng/mL epidermal growth factor (EGF; Sigma-Aldrich Cat# E5160-100UG), 1% insulin-transferrin-selenium (ITS; Thermo Scientific Cat# 51,500,056), and 1% penicillin/streptomycin (Thermo Scientific Cat# 15,140,122). The flask was incubated overnight in a humidified 37 °C incubator with 5% CO₂. The following day, secretory cells appeared as clustered “island”-like colonies adhered to the flask (Fig. 2A and B) as previously described by Karst and Drapkin [21] ideally reaching ~ 20–30% confluency. However, variability in tissue quality may result in lower viability and confluency. Ciliated cells usually do not adhere unless attached to secretory cells. Ciliated cells, being non-proliferative in 2D culture, typically disappear over time [21]. The medium was aspirated to remove non-adherent cells and debris. The adherent cells were gently rinsed with 1 mL PBS using a serological pipette. If debris was present over the cells, forceful rinsing was avoided to prevent dislodging viable secretory cells. After rinsing, the PBS was aspirated and replaced with 4 mL of fresh culture medium. The culture was maintained until cell confluency reached approximately 70%, which generally required 4–5 days, after which the cells were either subcultured or stored in liquid nitrogen for future use. The culture medium was refreshed every 24 h to remove debris and prevent pH fluctuations in the medium.

### Immunofluorescence

Trypsinized cells were washed with 1X PBS and fixed using paraformaldehyde (PFA, 4%). Fixed cells were air-dried on a clean glass slide. Permeabilization was carried out using 0.3% PBST (0.3% Triton X-100 in PBS) for 10 min. Cells were then blocked using PBST [0.1% Tween-20 and 1% Bovine Serum Albumin (BSA) in PBS] for 30 min. The cells were incubated overnight with primary antibodies- PAX8 (1: 100 dilution, Proteintech Cat# 10,336–1-AP, RRID:AB_2236705); Kininogen-1 (KNG-1) (1:100 dilution, Origin Diagnostics & Research Cat# OLK20692) and Annexin-V (1:100 dilution, Cell Signaling Technology Cat# 8555, RRID:AB_10950499) in PBST at 4 °C. The cells were incubated with the secondary antibody with goat anti-rabbit IgG H&L Alexa Fluor®488 (1:1000 dilution, Abcam Cat# ab150077, RRID:AB_2630356) at 37 ◦C for 1 h and then mounted using Fluoroshield™ with DAPI (Abcam Cat# ab104139a). The images were grabbed with the help of LAS X imaging software (Leica Application Suite X, Version 3.5.7.23225, RRID:SCR_013673) attached to fluorescence microscope (Axio Imager.A1, Zeiss, Germany). A minimum of 1000 cells were counted and the percentage cells with positive signal for PAX8 were calculated. hPFTSECs were imaged under 400 × magnification.

### Western Blot

For Western blotting, cultured hPFTSECs were treated with CC for 24 h and cells were lysed using cold RIPA lysis buffer (Sigma-Aldrich Cat# R0278). The suspension was centrifuged at 14,000 g for 15 min at 4 °C, and protein concentration was determined from the supernatant using BCA Protein estimation assay kit (Takara Bio Cat# T9300 A). Subsequently, 10 µg of protein lysate was loaded onto a resolving 12% SDS-PAGE and transferred to a methanol-activated PVDF membrane (Millipore Cat# IPVH00010) by using a semi-dry Trans blot apparatus (Bio-Rad). The membrane was blocked with 10% skimmed milk in TBST (0.1% TBS, 0.05% Tween 20) for 1 h at RT. The membrane was then incubated overnight with primary antibody Kininogen-1 (1:1000, Origin Diagnostics & Research Cat# OLK20692). After incubation with HRP-conjugated secondary antibody with goat anti-rabbit IgG-HRP (Santa Cruz Cat# sc-2004) at 37 ◦C for 2 h, protein bands were detected using a chemiluminescent reagent (Advasta Cat# K-12045-D20). GAPDH (ABclonal Cat# AC002) was used as a protein loading control.

### Fluorescence-activated Cell Sorting (FACS) Analysis

Cells were trypsinized, washed with 1X PBS, and fixed in 70% chilled ethanol. Samples were stored at 4 ◦C until use. Cells were permeabilized with 1X saponin solution for 30 min at room temperature (RT) on a rocking platform. Non-specific sites were blocked using 20% FBS in 1X Saponin (HiMedia Cat# RM405-100G) solution for 20 min at RT. Primary antibody (PAX8, Proteintech Cat# 10,336–1-AP, RRID:AB_2236705) was added at a 1:400 dilution in 1X saponin solution and incubated overnight at 4 °C on a rocker. After washing with 1X PBS, secondary antibody (goat anti-rabbit IgG H&L Alexa Fluor®488, Abcam Cat# ab150077, RRID:AB_2630356) was added at a 1:800 dilution in 1X saponin solution and incubated in the dark for 1 h at RT. The cells were washed with 1X PBS, resuspended in PBS, and events were acquired on an LSR II flow cytometer using a 488-laser line.

### Secretome Collection

A total of 0.75 × 10^6^ hPFTSECs at passage 3 were seeded in T-75 tissue culture flasks (Tarsons Cat# 950,020) and incubated at 37 °C for 6 h. Following incubation, the cells were treated with 5 µM and 10 µM CC for 24 h. CC (Sigma-Aldrich Cat# C6272-1G) stock solution was prepared using a 1:1 mixture of PBS and dimethyl sulfoxide (Sigma-Aldrich Cat# D4540-100ML). Following CC treatment, cells were washed with PBS and subjected to starvation by replacing the medium with DMEM F-12 without serum and growth factors for 4 h to induce protein secretion. This was followed by a 24 h incubation in serum-free DMEM F-12 medium supplemented with growth factors (EGF and ITS). Subsequently, the spent media/culture supernatants were collected and centrifuged for 10 min to get rid of any debris. Secretome samples of 10 mL were collected from each group across five independent trials, with samples obtained from five different patients.

### Protein Digestion

Equal volumes of protein lysate from each condition were taken and subjected to acetone precipitation overnight. After centrifugation, the pellet was resuspended with Triethylammonium bicarbonate buffer (TEAB), and the protein amount was estimated using the bicinchoninic acid (BCA) estimation method. Then, an equal amount of protein from each condition was reduced with dithiothreitol (DTT) at a final concentration of 10 mM for 20 min at 60 °C and alkylated with 20 mM iodoacetamide (IAA) and incubated for 20 min at room temperature in the dark. Trypsin at a ratio of 1:20 (enzyme to substrate) was added and incubated at 37 °C. The digested tryptic peptides were purified using Sep-Pak® C18 cartridges (Waters, MA, USA). The peptide estimation was carried out for each sample and dried using the speedvac. The dried peptides were then analyzed using LC–MS/MS [22].

### Mass Spectrometry Analysis

Mass spectrometry analysis was carried out using an Orbitrap Fusion Tribrid mass spectrometer (Thermo Fisher Scientific, Bremen, Germany) interfaced with Easy-nLC-1200 (Thermo Scientific) chromatography system. The peptide digests that had previously been dried were resuspended in 0.1% formic acid and loaded onto a trap column (Acclaim PepMap, 2 cm X 0.75 µ ID, 2 µm) by transferring from a 96-well LC plate. Peptides were separated by passing mobile phase B (0.1% FA in 80% ACN) in gradient mode for 120 min at 300 nL/min based on hydrophobicity in an analytical column (Acclaim PepMap, 50 cm X 0.75 µ ID, 2 µm). The data was acquired using an Orbitrap mass analyzer in data-independent acquisition mode (DIA) over a 120-min gradient. The percentage of mobile phase B was kept constant at 5% from 0 min, then gradually increased to 40% after 90 min, then to 100% after 15 min, and then kept constant for the next 15 min. Throughout the experiment, the temperature of the analytical column was kept at 45 °C. Peptides were ionized using a spray voltage of 2.1 kV and an ion transfer tube temperature of 275 °C. The positive precursor ions in the 350–1500 m/z range were detected in an Orbitrap at 120 K resolution, with a maximum injection time of 20 min. The RF level on the S-Lens was set to 55 throughout the acquisition. The precursor molecules in the 350–1500 m/z range were filtered in a quadrupole with 15 isolation windows and were fragmented at 35% HCD after the full MS scan. A normalized automatic gain control (AGC) target of 400% was used. The fragment ions within the m/z 145–2000 range were detected in an orbitrap at a resolution of 30 K. The precursor and fragment scans were acquired in profile and centroid mode, respectively [22].

### Data Analysis

LC–MS/MS raw data was searched against the Human RefSeq protein database (version 110) containing sequences with a common contaminant database using DIA-NN 1.8.1 [23]. Trypsin was chosen as the enzyme of choice, and a maximum of two missed cleavages were permitted. The oxidation of methionine and the acetylation of protein N-termini were designated as variable modifications, while the carbamidomethylation of cysteine was designated as a static modification. The data was compared with a false discovery rate (FDR) score threshold of 1% [23].

### Bioinformatics Analysis

Data obtained from DIA N–N was normalized based on the median and analyzed for differential abundance. Proteins were categorized as more abundant if they exhibited a fold change ≥ 1.5 and as less abundant if they exhibited a fold change ≤ 0.66, with statistical significance set at *p* < 0.05. Volcano plot, Heatmap, and Principal component analysis (PCA) were developed using R. Enrichment analysis of subcellular localization, molecular functions, and biological processes of differentially abundant proteins (DAPs) was carried out by g: profiler (https://biit.cs.ut.ee/gprofiler/gost). Pathway enrichment analysis was performed using g: profiler showing enriched Reactome and Kyoto Encyclopedia of Genes and Genome (KEGG) pathways. Protein–protein interaction analysis was performed using STRING (accessible at https://string-db.org/). Signal peptides were analyzed using the SignalP-5.0 online tool, accessible at (https://services.healthtech.dtu.dk/services/SignalP-5.0). Venn diagrams were generated using the Venny 2.1.0 tool (available at https://bioinfogp.cnb.csic.es/tools/venny/).

### Statistical Analysis

Statistical power analysis was conducted using GraphPad Prism 8.0 (GraphPad Software Inc., USA, RRID:SCR_002798), with the results expressed as mean ± SE. Differences between the control and test groups were evaluated through one-way ANOVA, with a *p*-value of less than 0.05 indicating statistical significance.

## Results

### Proteomic Analysis of CC-induced hPFTSEC Secretome Profiles

**Fig. 3:**
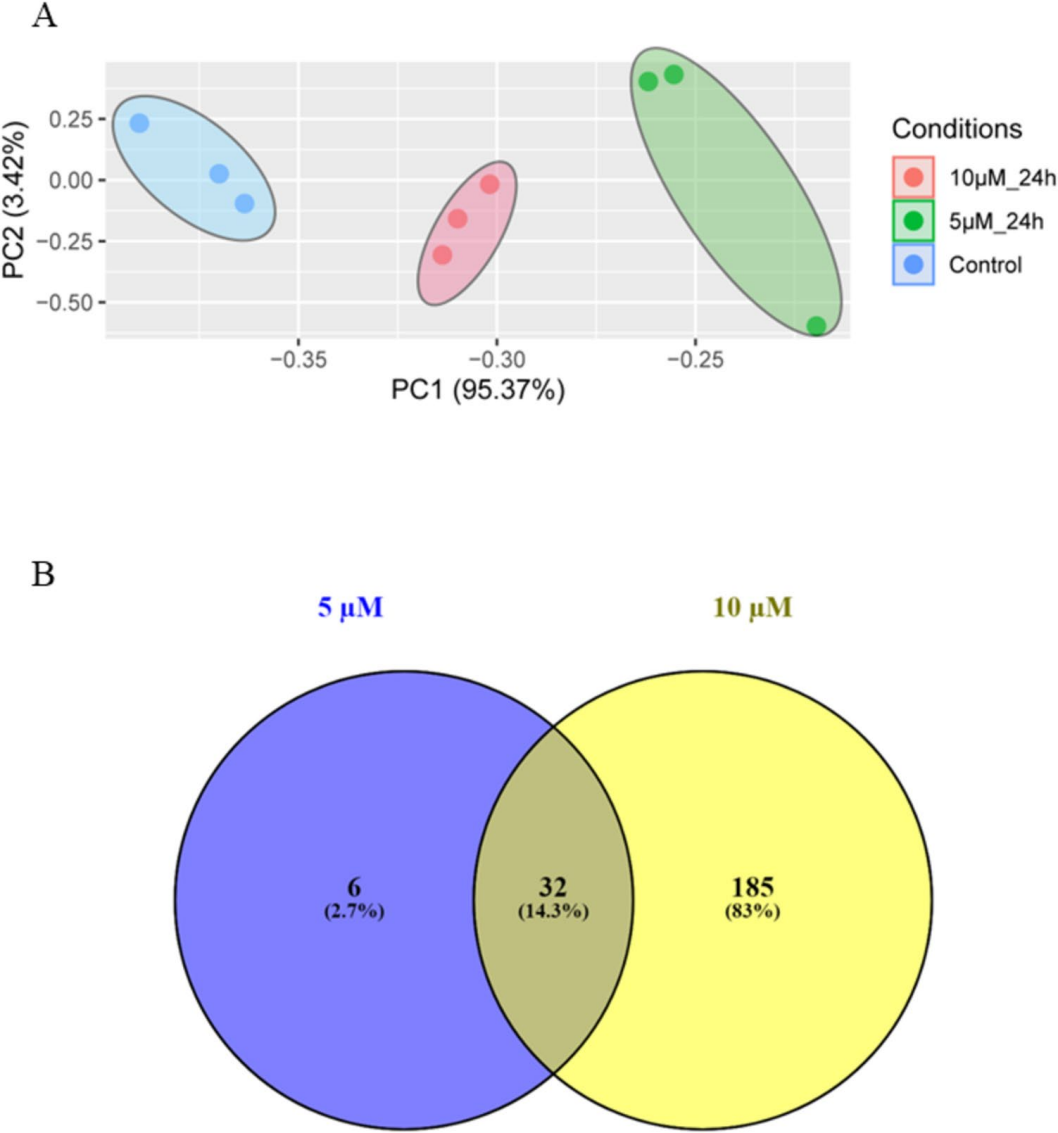
Proteins identified in CC-induced secretome profile of hPFTSEC by mass spectrometric method. **A** Principal component analysis (PCA) plot showing differential clustering of differentially abundant proteins (DAPs) in the secretomes collected from control and CC treated (5 and 10 µM) hPFTSECs. **B** Venn diagram depicting the DAPs and overlapping proteins secreted from hPFTSECs exposed to 5 and 10 µM CC

The secretory nature of hPFTSECs was confirmed by studying the PAX8 expression pattern in cultured cells from passage 1. We have observed that the typical cobblestone morphology [21] and secretory characteristics of hPFTSECs remain unchanged up to passage 5. Immunofluorescence data revealed that 93.57 ± 2.79%(*N = *3) of cells were positive for PAX8 expression (Fig. 2C), while FACS analysis revealed 99.2% PAX8-positive cells (Fig. 2D). The cultured PAX8-positive cells were exposed to CC for 24 h, followed by a 4 h starvation. Subsequently, the cells were incubated in growth factor-enriched medium for 24 h, after which secretomes were collected. We identified a total of 106,295 peptide spectrum matches (PSMs) and 11,076 peptides corresponding to 1549 proteins using an FDR cutoff of 1%. Principal component analysis (PCA) was carried out to analyze the protein profiles within the secretome samples. Furthermore, PCA of the identified proteins in the secretome samples highlighted distinct clustering among the control, 5 and 10 µM groups (Fig. 3A).

### Differentially Abundant Proteins in CC-induced Secretomes

To investigate the impact of CC on hPFTSEC secretome profiles, we analyzed differentially abundant proteins (DAPs) in each group after CC exposure. In the cells exposed to 5 µM CC, 39 proteins were significantly abundant (24 proteins with higher abundance, fold-change cutoff 1.5, *p*-value < 0.05 and 15 proteins with lower abundance, fold-change cutoff 0.66, *p*-value < 0.05). For the cells treated with 10 µM CC, 219 proteins were significantly abundant (123 proteins with higher abundance, fold-change cutoff 1.5, *p*-value < 0.05 and 96 proteins with lower abundance, fold-change cutoff 0.66, *p*-value < 0.05). The DAPs identified are summarized in Supplementary File 1. Table 1 summarizes the DAPs from the secretomes of hPFTSEC exposed to CC with specific roles in reproduction. The results from these two sets were compared and illustrated using a Venn diagram (Venny 2.1) to identify the number of DAPs in each group. This comparative analysis revealed that 185 proteins were uniquely identified in the 10 µM CC group, while six proteins were exclusive to the 5 µM CC exposure group. Additionally, 32 proteins were common to both groups (Fig. 3B).

**Table 1:** The differentially abundant proteins from secretomes of hPFTSECs exposed to clomiphene citrate with specific roles in reproduction

S. no	Function	Candidate Proteins
1	Pregnancy	IGFBP2, IGFBP5, IGFBP7, STC2, MMP2, ITGA2, PZP, CALR, FBLN1
2	Embryonic morphogenesis	COL5 A1, FBN1, LAMA3, LAMB3, PTK7, FN1, CCN1, COL4 A2, COL6 A1, INHBA, LAMB1, SDC4, COL12 A1, MMP2, RBP4, ITGA2
3	Embryo implantation	IGFBP7, STC2, MMP2, CALR, FBLN1
4	Embryo development	COL1 A1, COL3 A1, COL5 A1, FBN1, LAMA3, LAMB3, PTK7, FN1, CCN1, COL4 A2, COL6 A1, INHBA, LAMB1, SDC4, NRP2, COL12 A1, RPL13, RBP4, MMP2, ITGA2, RTN4
5	Reproductive system development	C3, SERPINE2, INHBA, MMP2, IDH1, RBP4, HSPA5
6	Regulation of embryonic development	LAMA3
7	Maternal process involved in female pregnancy	STC2
8	Sperm-egg recognition	VDAC2, CCT7
9	Fertilization	MFGE8, VDAC2, CCT7
10	Embryo development ending in birth or egg hatching	COL1 A1, COL3 A1, PTK7, CCN1, SDC4, RPL13, RBP4, RTN4
11	Binding of sperm to zona pellucida	VDAC2, CCT7
12	Female gamete generation	INHBA, MMP2
13	Gamete generation	INHBA, MMP2, KRT9, CALR
14	Ovulation	A2 AP-A
15	Peri-implantation	PXDN
16	Healthy pregnancy	DPP1, STC2, IGFBP
17	Pre-term birth	HSP70
18	Miscarriage and pregnancy loss	PRDX2, PZP, ANXA5, VDBP, POSTN-2, POSTN-2
19	Ectopic pregnancy	FSTL1, FSTL3 LGALS3BP, TIMP1, TIMP2, MMP2, MMP-8, DCN-A, CDH13-2, ACTA1
20	Sperm interaction	KRT, ANXA5
21	Sperm migration in the oviduct	CANX-D
22	Maturation of sperm membrane proteins	CRT
23	Sperm survival	HSPA6
24	Sperm motility	KNG1, CST3
25	Sperm liquefaction	KLK6-A
26	Capacitation	MIF
27	Capacitation and acrosome reaction	CST3
28	Sperm-egg binding	FN8
29	Estrogen-dependent gene expression	H3 C15, H4 C15, H2 AC19, PTGES3, CTSD
30	ESR-mediated signaling	MMP3, MMP7, MMP2, H3 C15, H4 C15, H2 AC19, PTGES3, CTSD
31	Estrogen signaling pathway	MMP2, KRT9, KRT10, KRT17, KRT14, CTSD, HSP90B1, HSPA1 A
32	Extra-nuclear estrogen signaling	MMP3, MMP7, MMP2
33	Response to progesterone	THBS1
34	Progesterone secretion	INHBA
35	Progesterone-mediated oocyte maturation	INS
36	Regulation of anti-Mullerian hormone signaling pathway	DKK3
37	Steroid binding	GC, VDAC2, VDAC1
38	Response to steroid hormone	COL1 A1, IGFBP2, IL6, IGFBP7, ACTA1, CCN2, LOX, THBS1, ATP5 F1 A, IDH1, PTGES3, CALR, ANXA3, HSPA1 A
39	Response to corticosteroid	COL1 A1, IGFBP2, IL6, IGFBP7, CCN2, ATP5 F1 A, ANXA3, IL6
40	Regulation of steroid biosynthetic process	DKK3, IGFBP7, APOE, DKK3, AKR1B1
41	Ovarian cancer and fallopian tube lesions	CTSD, KRT, H2, SPOCK1, SPON1, HSP10, TF, ILK1, CKAP4, LAMP1, RTN4E, JUP, CDH11, COL, LGALS3BP, TGFBI, HTRA1, CD44, MMP2, CDH13-2, TMSB4X, PTGES3B

**Fig. 4:**
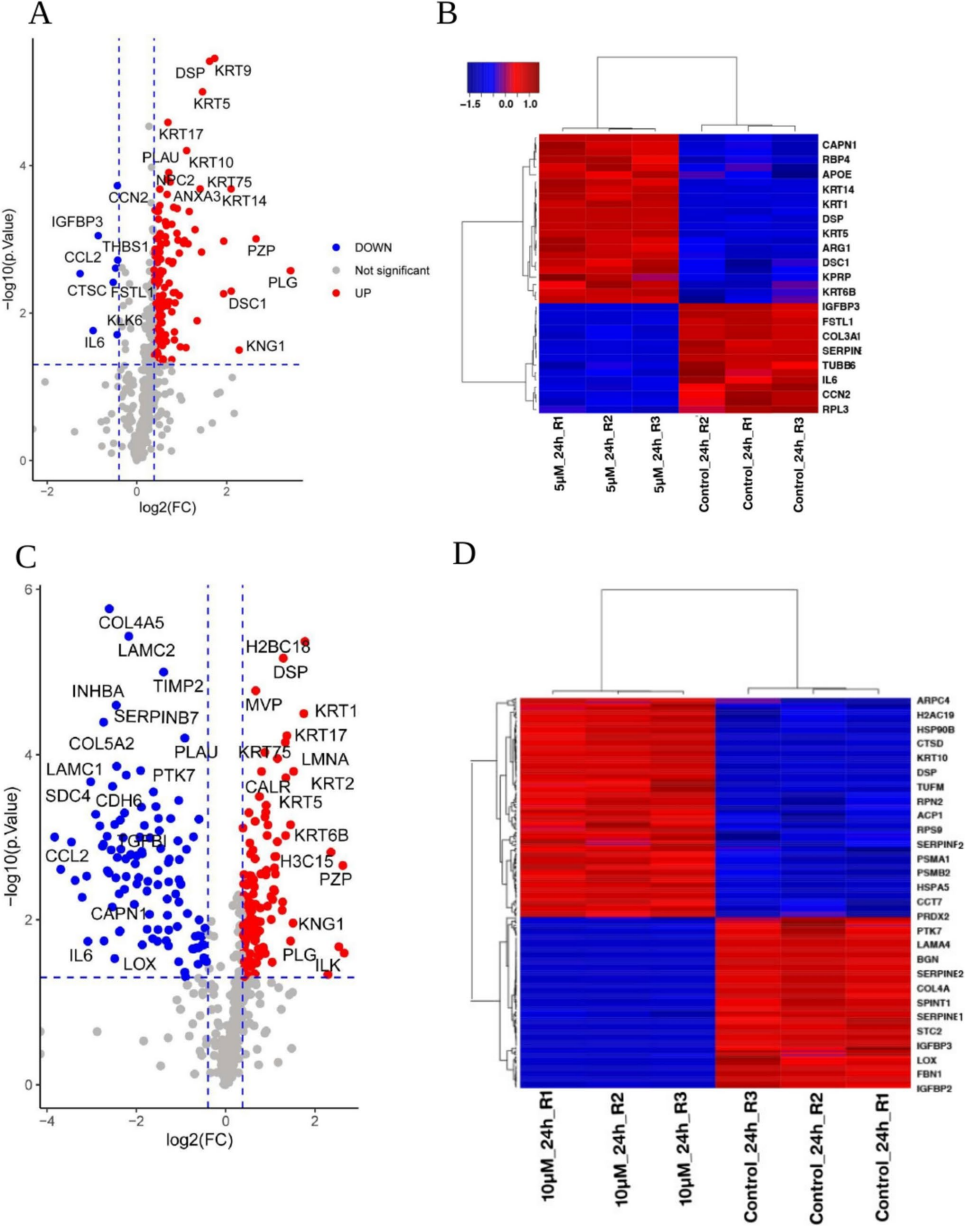
Protein abundance in the hPFTSEC secretome following CC exposure. **A** Volcano plot showing the DAPs [more abundant (red) and less abundant (blue)] secreted when exposed to 5 µM CC. **B** Heat map depicting the differences in the protein abundance between triplicates of proteins (R1, R2, and R3) represent triplicates for each condition) secreted when exposed to 5 µM CC. **C** Volcano plot showing the DAPs [more abundant (red) and less abundant (blue)] secreted when exposed to 10 µM CC. **D** Heat map depicting the differences in the protein abundance between triplicates of proteins (R1, R2, and R3 represent triplicates for each condition) secreted when exposed to 10 µM CC

The distribution of the DAPs based on fold change was analyzed using clustering analysis. Volcano plots were generated to visualize proteins based on their fold changes and statistical significance as determined by a student’s t-test (Fig. 4A and C). The corresponding heatmap illustrates the variations in protein abundance across triplicates within each group (Fig. 4B and D). Following exposure to CC, higher abundance of keratins (KRT1, KRT2, KRT6B, KRT9, KRT14, KRT17), pregnancy zone protein (PZP), desmocollin-1 (DSC1), hornerin (HRNR), different histones (H2BC18, H3 C15) were observed. Conversely, proteins such as C–C motif chemokine 2 (CCL2), insulin-like growth factor-binding protein 3 isoform b (IGFBP3), kallikrein-6 (KLK6), thrombospondin-1 (THBS1), interleukin-6 (IL6), dipeptidyl peptidase 1 (DPP1), follistatin-related protein 1 (FSTL1), syndecan-4 (SDC4), laminin subunit gamma-1 (LAMC1), tissue inhibitor of metalloproteinases 2 (TIMP2), and serine protease inhibitor B7 (SERPINB7) were found to be less abundant upon CC treatment.

**Fig. 5:**
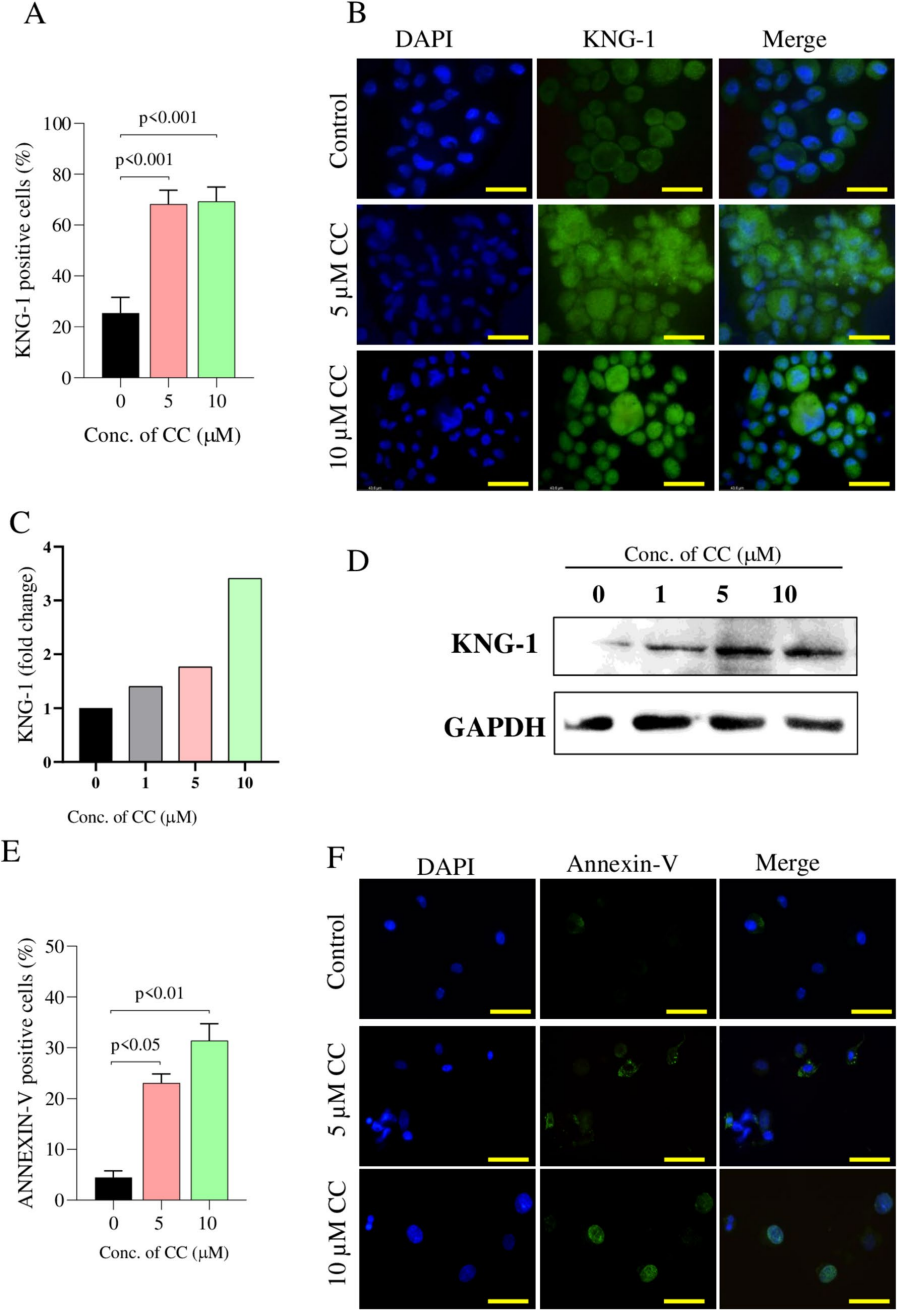
Validation of proteomic data in hPFTSECs. **A** Percentage of hPFTSECs expressing Kininogen-1 protein studied by immunofluorescence technique. **B** Representative images of hPFTSCEs expressing Kininogen-1 protein by immunofluorescence (400 × magnification). The scale bar represents 50 µm. The data is represented as Mean ± SE (*N = *3). **C** Quantification of Kininogen-1 protein levels, normalized to GAPDH and expressed as fold change relative to untreated control (*N = *1). **D** Representative Western blot showing Kininogen-1 protein expression in hPFTSECs treated with CC (0, 1, 5 and 10 µM) for 24 h. GAPDH was used as a loading control. **E** Percentage of hPFTSECs expressing Annexin-V assessed by immunofluorescence. **F** Representative images of hPFTSECs expressing Annexin-V by immunofluorescence (400 × magnification)**.** The scale bar represents 50 µm. The data is represented as Mean ± SE (*N = *3)

Two proteins that exhibited significant higher abundance were further confirmed in hPFTSECs treated with 5 and 10 µM CC. Immunofluorescence analysis revealed that the expression of Kininogen-1, one of the top three most abundant proteins in the secretome, was significantly increased (*p* = 0.0213, fold change 5.8) at both 5 and 10 µM concentrations (Fig. 5A and B). Western blot analysis further confirmed this upregulation, showing fold change increases of 1.4, 1.8, and 3.4 at CC concentrations of 1, 5, and 10 µM, respectively (Fig. 5C and D). Similarly, Annexin-V expression (*p* = 0.0017, fold change 2.2), assessed by immunofluorescence, was significantly higher at both 5 and 10 µM concentrations (Fig. 5E and F), validating our proteomic results.

### Gene Ontology Analysis

**Fig. 6:**
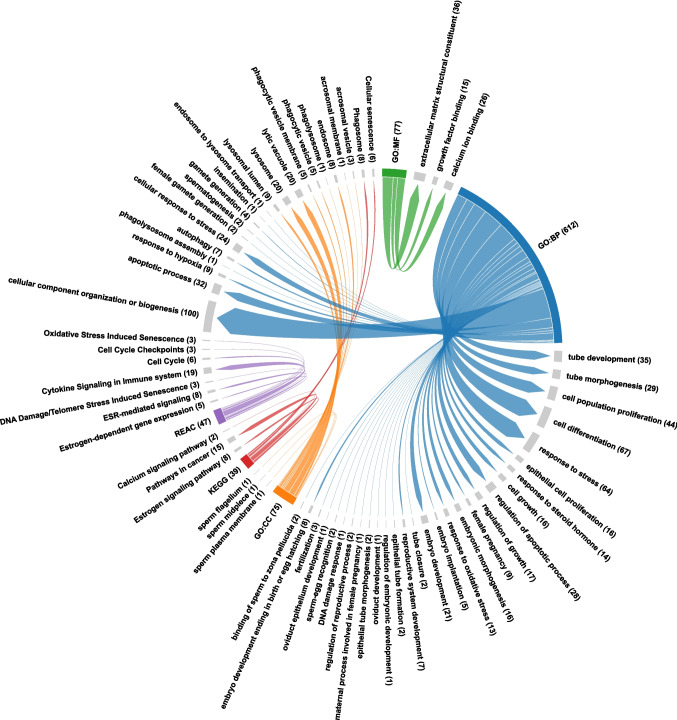
Gene ontology analysis of secreted proteins identified in the present study. Gene ontology enrichment analysis of the DAPs represented in molecular function, cellular components, biological process and KEGG/REAC. The number of contigs for each term is represented within colored panels

GO analysis revealed that the secreted proteins are involved in various stress pathways, including cellular response to stress, response to hypoxia, response to oxidative stress, and cellular response to chemical stress, indicating a CC-induced stress response. Further analysis revealed that the DAPs are involved in processes such as autophagy, phagolysosome assembly, endosome-to-lysosome transport, sperm-egg recognition, sperm binding to the zona pellucida, fertilization, embryo implantation, embryo development, maternal processes involved in pregnancy, and regulation of the reproductive process. This indicates that CC might interfere with the primary role of the fallopian tube in creating an optimal environment for fertilization and peri-implantation. Furthermore, DAPs were found to be involved in processes related to DNA damage, apoptosis, cellular senescence and cancer pathways. It was also confirmed that several secreted proteins are localized to cellular components, including the phagosome, lysosome, phagolysosome, phagocytic vesicle, lytic vacuole, and endosome. Further, various sperm cellular components were identified in the secretome, including the sperm plasma membrane, acrosomal membrane, acrosomal vesicle, sperm midpiece, and sperm flagellum. This suggests CC might trigger a phagocytic response towards spermatozoa in the fallopian tube microenvironment (Fig. 6).

### Pathway Enrichment

CC demonstrates both estrogen agonist and antagonist properties. However, its agonistic effects are typically observed only under conditions of significantly low endogenous estrogen levels. Otherwise, CC primarily functions as a competitive estrogen antagonist. KEGG and Reactome analyses showed enrichment in estrogen and ESR-mediated signaling pathways (Fig. 6), while transcription factor enrichment of CC-treated secretomes identified four key transcription factors associated with ESR2/ERβ (Supplementary File 4). The findings suggest that CC, a structural analog of estrogen, might influence these pathways through a regulatory role of ERβ in modulating the observed secretome profile. Additionally, pathway analysis identified processes related to progesterone response and secretion, steroid hormone response, regulation of steroid biosynthesis, and anti-Mullerian hormone signaling, suggesting that CC treatment might disrupt the hormonal balance in the fallopian tube microenvironment. The Reactome analysis further identified that these proteins participate in the cell cycle and DNA damage checkpoints, suggesting that CC induces DNA damage, apoptosis, and senescence (Fig. 6).

### Network Analysis

**Fig. 7:**
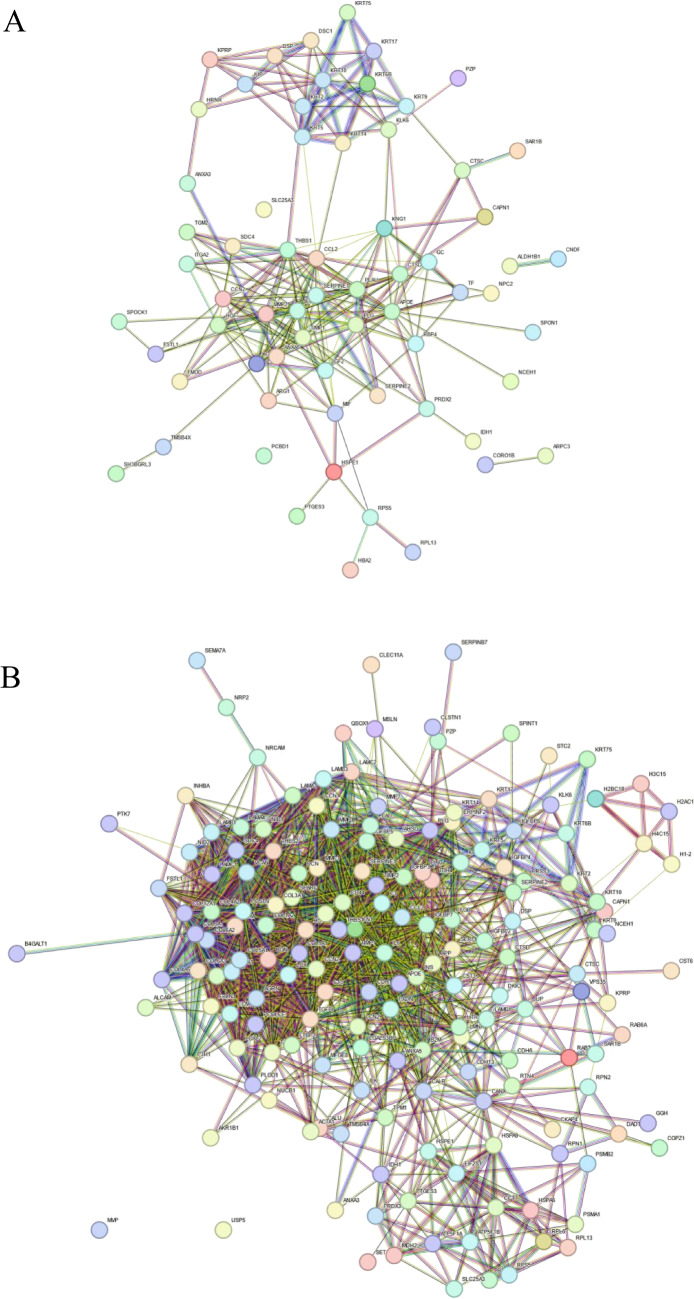
Protein–protein interaction network analysis of DAPs. **A** hPFTSECs exposed to 5 µM CC and, **B **hPFTSECs exposed to 10 µM CC

The DAPs were also subjected to network analysis using the STRING database. The protein–protein interaction network was constructed separately for 5 µM and 10 µM groups by taking DAPs from the two groups respectively. The protein–protein interaction network is shown in Fig. 7. Network analysis from the DAPs in the 5 µM group revealed a major cluster comprising proteins involved in apoptosis, pregnancy, tube development, cellular component organization, and estrogen signaling pathway and a minor cluster comprising mainly keratins and PZP. Network analysis from the DAPs in the 10 µM group revealed a major cluster comprising multiple proteins involved in apoptosis, cellular compartment organization, tube development, pregnancy, embryo implantation, and embryo development. Three minor clusters were also identified, one of which mostly included keratins which are involved in the estrogen signaling pathway (KRT9, KRT10, KRT17, KRT14).

### SignalP Analysis

Secretory proteins typically feature positively charged short sequences at their N-terminal ends, aiding their secretion upon cleavage [24]. Signal peptides play a crucial role in directing the protein for secretion via the classical secretory pathway. In proteomics studies focused on the secretome, SignalP primarily identifies proteins with signal peptides. In this study, proteins containing signal peptides were identified using the SignalP-5.0 online tool to determine the proportion of secreted proteins. From our analysis using SignalP, we identified 7 proteins that were found to harbor signal peptides. Proteins containing a cleavable signal peptide sequence were classified as classical secreted proteins, while the remaining were categorized as non-classical secreted proteins. A detailed list of these proteins can be found in Supplementary File 2. Notably, proteins such as HPX, FAP, NXPE3, ITGA3, LAMC1, FSTL1, and SPARC were identified to contain signal peptides. These proteins exhibit diverse functions in cellular processes such as tissue remodeling, adhesion, and inflammation.

## Discussion

In this study, for the first time we report the CC-induced changes in the secretome profile of hPFTSECs. After confirming the secretory nature of the cells through PAX8 expression, proteomic analysis of the hPFTSECs revealed alteration in pathways and candidate proteins associated with various processes such as estrogen signaling, ovulation, embryo development, implantation, pregnancy complications, ectopic pregnancies, sperm functions, apoptosis, senescence, cell cycle regulation, DNA damage, ovarian cancers, etc. The proteomic results were validated by confirming the expression of two significantly more abundant proteins, Kininogen-1 and Annexin-V, in hPFTSECs treated with 5 and 10 µM CC. Further, our comprehensive literature review identified specific proteins in the secretomes associated with specific roles in reproduction and fertilization process.

Previous studies have indicated that although CC has a high ovulation induction rate, the pregnancy rate remains quite low [2, 3]. As observed in our study, the CC-induced alteration in the secretome profile may lead to subtle changes in the microenvironment of the fallopian tube, possibly leading to reduced sperm motility and viability, as well as interfering with the prerequisites for fertilization, including capacitation and the acrosome reaction. Previous studies have demonstrated that small peptides secreted by human fallopian tube cells help to maintain sperm motility, while cell membrane proteins from these cells protect the spermatozoa from oxidative damage [25, 26]. Coy et al*.* (2010) reported that porcine oviductal fluid can improve sperm viability in vitro [27]. Similarly, enriched protein fractions from oviductal fluid have been reported to preserve buffalo sperm motility and viability during cryopreservation [28]. It has been reported that sperm binding to the fallopian tube epithelium helps in maintaining their fertilizing ability during storage, and sperm incubated with fallopian tube epithelial cells in vitro exhibit prolonged survival compared to those in culture medium alone [29]. GO analysis of the secretome identified various sperm cellular components, such as the sperm plasma membrane (HSP90B1), acrosomal membrane (MFGE8), acrosomal vesicle (MFGE8, VDAC2, CALR), sperm midpiece (VDAC2), and sperm flagellum (VDAC2), suggesting that spermatozoa interact with these proteins secreted in response to CC exposure.

Studies have reported that during pre-ovulatory intervals, the fallopian tube employs a strategy of delaying complete sperm capacitation, which helps to store sperm in a non-capacitated state in the caudal isthmus for a prolonged period [30]. Suppression of complete capacitation serves as a strategic storage mechanism to maintain sperm viability and functionality throughout the pre-ovulatory interval, preventing premature loss of energy and enzymatic activity, ensuring that sperm remains viable and ready for fertilization when ovulation occurs [30]. Several abundant proteins from the secretome collected from CC exposed hPFTSCs have been reported to be associated with various sperm functions. These include keratin, type II cytoskeletal 5 (KRT5), which is known to interact with sperm-associated antigen 5 (SPAG5) [31]; calreticulin (CRT), which is reported to aid in the maturation of sperm membrane proteins [32]; calnexin (CANX), which plays a role in sperm migration in the fallopian tube as well as in the maturation of sperm membrane proteins [32]; annexin A5 (ANXA5), which is recognized for its role in sperm interaction as well as in the binding of spermatozoa to the fallopian tube [33] and macrophage migration inhibitory factor (MIF), which has adverse effects on sperm motility when present in elevated levels [34]. Further, we identified several less abundant proteins in the hPFTSEC secretome that are associated with various sperm functions. Heat shock protein 6 (HSPA6), which supports sperm survival, kallikrein (KLK), which enhances sperm motility [35], and cystatin-C (CST3), which maintains sperm fertilizing ability by preventing premature capacitation and acrosome reaction while also promoting motility [36] were among the less abundant proteins in the secretome of CC treated hPFTSECs. The higher abundance of proteins involved in sperm capacitation and membrane maturation, and lower abundance of proteins essential for motility and sperm integrity following CC exposure, suggests premature capacitation and a loss of sperm fertilizing ability, potentially leading to reduced fertility.

Even though controversial, previous studies have demonstrated that fallopian tube epithelial cells have the ability to phagocytose spermatozoa [37–39]. GO analysis confirms that CC induces a phagocytic response in the fallopian tube microenvironment by involving proteins in phagocytosis-related processes and localizing spermatozoa to cellular components such as phagosomes, lysosomes, and endosomes (Fig. 6; Supplementary File 3). An enhanced phagocytic response induced by CC might affect sperm survival and/or embryo development in fallopian tube which could potentially explain the low pregnancy rates observed in CC-induced fertility programs. However, further studies are needed to confirm this. Further, GO analysis identified proteins involved in sperm-oocyte recognition (VDAC2, CCT7). The lower abundance of the protein fibronectin 8 (FN8), which is reported to be associated with the initiation of sperm-oocyte binding observed in the secretome of CC treated hPFTSECs [40] further confirms that CC can interfere with the sperm-oocyte interaction. In addition, the lower abundance of proteins involved in fertilization (MFGE8, VDAC2, CCT7), embryo development (INHBA, NRP2, MMP2, ITGA2, RTN4, etc.) and initiation of pregnancy (IGFBP2, PZP, CALR, STC2, FBLN1, etc.) further indicates the alteration in fallopian tube microenvironment affecting the normal fertilization and embryo development process.

Moderately high risk of spontaneous abortions [7–9] and risk of abnormal pregnancies [10] in women undergoing fertility treatment with CC has been reported earlier. The higher abundance of proteins associated with miscarriage, recurrent pregnancy loss and preterm birth such as pregnancy zone protein (PZP) [41], annexin A5 (ANXA5) [42], vitamin D-binding protein (VDBP) [43] and heat shock protein-70 (HSP70) [44] in the secretome of CC treated hPFTSECs of our study is consistent with earlier studies showing a higher rate of abortion and pregnancy loss in CC-induced pregnancies compared to spontaneous conceptions [8, 45]. Periostin (POSTN) [46], known to be decreased in individuals experiencing spontaneous pregnancy loss, showed lower abundance following CC treatment. Subsequently, proteins that have been reported for creating a conducive environment in the fallopian tube for conception, peri-implantation, and are essential for establishing and maintaining a healthy pregnancy, such as insulin-like growth factor-binding protein (IGFBP) [47], Peroxidasin (PXDN) [48], dipeptidyl peptidase (DPP) [49] and stanniocalcin (STC) [50] were less abundant in the secretome collected from CC-treated hPFTSECs.

Studies have further indicated that CC therapy induces apoptosis in the epithelial cells of the rat oviduct [51] and causes chromosomal abnormalities in human lymphocytes [52]. Agreeing with the literature, we observed an altered abundance of proteins and pathways associated with DNA damage, DNA damage checkpoints, apoptosis, atresia, and senescence in the CC-treated secretomes (Fig. 6; Supplementary File 3). Apoptosis and DNA damage might impair the fallopian tube epithelium and result in defective transport, potentially leading to ectopic pregnancies. CC treatment has been documented to be associated with higher incidence of ectopic pregnancy in humans [4–6, 53–55]. Proteins that have previously been reported to be reduced in cases of ectopic pregnancy including follistatin (FST) [56], galectin-3 (LGALS3) [57], metalloproteinase inhibitor 2 (TIMP2) [58, 59], and decorin (DCN) [60] were found to be less abundant in the CC-treated secretomes.

Based on the recent evidence, the fallopian tube is recognized as the primary site of origin for the majority of high-grade serous ovarian carcinomas (HGSOCs) [61–63]. In the CC-treated hPFTSECs-secretome, we observed a significant higher abundance of proteins associated with HGSOCs and fallopian tube lesions, which includes cathepsin D (CTSD) [64], keratins (KRT5, KRT14, KRT17) and histones (H2B, H4) [65, 66], testican-1 (SPOCK1) [67], 72 kDa type IV collagenase 2 (MMP2) [68], insulin-like growth factor II (IGF2) [69], spondin-1 (SPON1) [70], thymosin beta-4 (TMSB4X) [71], prostaglandin E synthase (PTGES) [72], 10 kDa heat shock protein (HSP10) [73], serotransferrin (TF) [74], integrin-linked protein kinase (ILK) [75], cytoskeleton-associated protein (CKAP) [76], lysosome-associated membrane glycoprotein 1 (LAMP1) [77], reticulon (RTN) [78], and junction plakoglobin (JUP) [79]. Further, lower abundance was observed for various proteins such as cadherin (CDH) [80], collagens (COL1, COL3), galectin-3-binding protein (LGALS3BP) [81], transforming growth factor-beta-induced protein ig-h3 (TGFBI) [82], serine protease HTRA1 (HTRA1) [83] and C44 antigen (CD44) [84], as observed in HGSOCs. Furthermore, KEGG analysis showed enrichment in cancer pathways, supporting the long-held hypothesis that HGSOCs might originate from the fallopian tube epithelium.

## Conclusions

The current investigation has revealed novel insights in understanding the CC-induced alteration in the fallopian tube microenvironment and its possible impact on abnormal fertilization/pregnancy outcomes. CC-induced changes in the fallopian tube secretome profile appear to disrupt pathways critical for sperm-oocyte interaction, sperm functionality, early pregnancy maintenance, embryo development and potentially contribute to lower pregnancy rates and complications, including ectopic pregnancy, findings that hold significant clinical implications. While the changes induced by CC might be temporary and the fallopian tube cells could return to normal function after discontinuation of treatment, the long half-life (~ 7 days) and poor clearance rate (~ 6 weeks) of CC [85] suggest it could have a potential sustained impact on the cells and the microenvironment. However, further studies are necessary to understand the impact of CC on the ciliary cells of the fallopian tube, which play a crucial role in sperm, oocyte and embryo transport. Further, the altered proteomic profile of CC treated cells hints towards the possible association of ovulation induction drugs with ovarian cancer, which needs to be studied further.

## Supplementary Information

Below is the link to the electronic supplementary material.

Supplementary file1 (XLSX 67 KB)

Supplementary file2 (XLSX 11 KB)

Supplementary file3 (XLSX 540 KB)

Supplementary file4 (XLSX 9 KB)

## Data Availability

Manuscript data are deposited in the PRIDE database under accession code PXD057963, it will be made public once the manuscript is accepted/published. Supplementary files will be accessible via DOI: 10.6084/m9.figshare.27952620 and 10.6084/m9.figshare.28829222 upon acceptance/publication of the manuscript. All other data generated or analyzed during this study are included in this published article (and its Supplementary Information files).
